# M1 macrophage-related gene model for NSCLC immunotherapy response prediction: Gene model for NSCLC immunotherapy response prediction

**DOI:** 10.3724/abbs.2023262

**Published:** 2024-02-21

**Authors:** Sifan Wu, Qiqi Sheng, Pengjun Liu, Zhe Jiao, Jinru Lv, Rong Qiao, Dongkun Xie, Zanhan Wang, Jiamei Ge, Penghui Li, Tiaoxia Wei, Jie Lei, Jieyi Fan, Liang Wang

**Affiliations:** 1 State Key Laboratory of Cancer Biology Department of Medical Genetics and Developmental Biology Fourth Military Medical University Xi’an 710032 China; 2 Department of Thoracic Surgery the Second Affiliated Hospital of Air Force Medical University Xi’an 710038 China; 3 Department of Aerospace Medicine Fourth Military Medical University Xi’an 710032 China

**Keywords:** M1 macrophage, NSCLC, immune infiltration, immunotherapy, prognosis prediction model

## Abstract

Patients diagnosed with non-small cell lung cancer (NSCLC) have a limited lifespan and exhibit poor immunotherapy outcomes. M1 macrophages have been found to be essential for antitumor immunity. This study aims to develop an immunotherapy response evaluation model for NSCLC patients based on transcription. RNA sequencing profiles of 254 advanced-stage NSCLC patients treated with immunotherapy are downloaded from the POPLAR and OAK projects. Immune cell infiltration in NSCLC patients is examined, and thereafter, different coexpressed genes are identified. Next, the impact of M1 macrophage-related genes on the prognosis of NSCLC patients is investigated. Six M1 macrophage coexpressed genes, namely, *NKX2-1*, *CD8A* , *SFTA3*, *IL2RB*, *IDO1*, and *CXCL9*, exhibit a strong association with the prognosis of NSCLC and serve as effective predictors for immunotherapy response. A response model is constructed using a Cox regression model and Lasso Cox regression analysis. The M1 genes are validated in our TD-FOREKNOW NSCLC clinical trial by RT-qPCR. The response model shows excellent immunotherapy response prediction and prognosis evaluation value in advanced-stage NSCLC. This model can effectively predict advanced NSCLC prognosis and aid in identifying patients who could benefit from customized immunotherapy as well as sensitive drugs.

## Introduction

The primary cause of cancer death in the world is lung cancer [1]. Non-small cell lung cancer (NSCLC), as the most common pathologic type of lung cancer, has a 5-year survival rate below 15% [ 2, 3]. For NSCLC, systemic chemotherapy has been the standard of management, but it provides only modest benefits [4]. Accumulating evidence and our previous studies indicated that immune checkpoint inhibitors (ICIs) represented by anti-programmed cell death ligand 1 (PD-L1) inhibitors are novel promising neoadjuvant therapies for advanced-stage NSCLC and have brought some success to the treatment of NSCLC [ 5, 6]. However, the objective response rate to ICIs in NSCLC has been reported to be less than 20%, indicating that not all NSCLC patients benefit from immunotherapy [ 7, 8]. Thus, identifying patients who can specifically benefit from immunotherapy to maximize the effectiveness is of great significance for the precise treatment of NSCLC.

The tumor immune microenvironment (TIM) plays an important role in the development and spread of NSCLC [ 9‒ 11]. Among all immune cells, tumor-associated macrophages (TAMs) constitute the predominant cellular component in the NSCLC tumor microenvironment (TME), which can contribute to NSCLC development by mediating immunosuppression, promoting proliferation and metastasis, and even exerting drug resistance [ 12‒ 14]. Based on environmental cues, TAMs can exhibit tumoricidal M1-like or protumor M2-like macrophage phenotypes [ 15, 16]. M1-like TAMs can display antitumor capability by phagocytosis, the production of immunostimulating factors, such as IL-12 and TNFα, and the initiation of the Th1 antitumor response, which can then significantly enhance the therapeutic effects of immunotherapy in NSCLC [ 17, 18]. In contrast, M2-like TAMs can recruit different types of immunosuppressive cells, such as regulatory T cells (Tregs) and myeloid-derived suppressor cells (MDSCs), inhibit T-cell activation and proliferation, and promote angiogenesis by secreting many proangiogenic factors [19]. The contribution of M1 macrophages to NSCLC progression and the mechanisms by which M1 macrophages mediate the response to immunotherapy remain unclear. Thus, considering the critical role of M1 macrophages in inhibiting tumor development and enhancing the immunotherapy response, whether phenotype and functional markers correlated with M1 macrophage infiltration can potentially be utilized to predict the immunotherapy response in NSCLC should be investigated.

In the present study, we hypothesized that expression of genes related to M1 macrophage infiltration in NSCLC might reveal the makeup of the TME, reflect immune cell infiltration, and thereby function as an immunotherapy response predictor for further precise NSCLC treatments. To validate this hypothesis, we investigated the TME landscape of NSCLC and demonstrated the relationship between immunotherapy response and M1 macrophage infiltration. Thereafter, a novel model for predicting NSCLC immunotherapy response was developed. The results indicated that the model based on six genes, namely, *NKX2-1*, *CD8A*, *IL2RB*, *IDO1*, *SFTA3*, and *CXCL9*, was able to accurately predict NSCLC prognosis and immunotherapy response. The expressions of these six genes were validated in the TD-FOREKNOW NSCLC clinical trial.

## Materials and Methods

### Data sources and preprocessing

NSCLC patients’ bulk RNA sequencing profiles and clinical data, including sex, treatment arm, histology, overall survival, and therapeutic response, were retrieved from the OAK [4] and POPLAR [20] programs in the EGA archive ( https://ega-archive.org/). The OAK and POPLAR program comprised randomized clinical trials of immunotherapy (atezolizumab) versus chemotherapy in NSCLC, and they represent the largest transcriptional collection compiled in NSCLC under these circumstances to date. After filtering, 891 advanced-stage NSCLC patients treated with either immunotherapy or chemotherapy were included in this research. NSCLC patients treated with atezolizumab (PD-L1 inhibitor) and best recorded overall response evaluated as disease progression (PD), complete response (CR), or partial response (PR) were set as the training dataset, and the rest were set as the test dataset. The external test set was downloaded from The Cancer Genome Atlas (TCGA) database, and the advanced stage (stage III/IV) of lung adenocarcinoma was included ( *n*=265). The clinical information is summarized in Table 1 and Supplementary Table S1.

**Table:** **Table 1** Characteristics of patients included in this study

Characteristics	Training set ( *n*=254)	Test set ( *n*=637)
Gender		
Female	78 (30.7%)	251 (39.4%)
Male	176 (69.3%)	386 (60.6%)
Treatment		
Docetaxel	0 (0.0%)	452 (71.0%)
MPDL3280A	254 (100.0%)	185 (29.0%)
BOCR		
CR	5 (2.0%)	0 (0.0%)
NE	0 (0.0%)	22 (3.5%)
PD	193 (76.0%)	150 (23.5%)
PR	56 (22.0%)	58 (9.1%)
SD	0 (0.0%)	344 (54.0%)
Unknown	0 (0.0%)	63 (9.9%)
Histology		
Non-squamous	188 (74.0%)	441 (69.2%)
Squamous	66 (26.0%)	196 (30.8%)

Clinicopathological characteristics of NSCLC patients from the OAK and POPLAR projects. PD, disease progression; SD, disease stable; PR, partial response; CR, complete response; NE, not evaluated.

### Immune cell infiltration

The R package “IOBR” was used to perform the ESTIMATE algorithm [21]. The function “deconvo_tme()” was then used to compute the score of 22 kinds of immune cell infiltration based on CIBERSORT [22]. QuanTIseq [23] was employed to compute the composition of 10 major kinds of cells present in the TME. xCell [24] was used for immune cell infiltration analysis.

The R packages “ggbeeswarm” and “ggplot2” were utilized to visualize the immune cell landscape in the form of both beeswarm boxplots and bar plots.

### Screening of M1 macrophage-related genes

Analysis of genes associated with M1 macrophage infiltration was performed based on the extent of M1 macrophage infiltration by CIBERSORT and gene expression data using R packages “tidyverse” and function “cor.test()”. Thereafter, the R packages “ggExtra” and “cowplot” were used to plot the correlation graphs.

### Enrichment pathway analysis

The gene symbol was converted to EntrezID using the R package “org.Hs.eg.db” for enrichment analysis. Gene Ontology (GO) analysis was performed by the R package “clusterProfiler” to explore various biological processes, molecular functions, and cellular components of the genes. Kyoto Encyclopedia of Genes and Genomes (KEGG) analysis was conducted on the website tool Metascape ( http://metascape.org) [25].

### Model construction

Based on the gene expression profile and clinical information, the R packages “survminer” and “survival” were utilized to perform Kaplan-Meier survival analysis. Thereafter, the genes were further determined by univariate Cox regression analysis. A forest plot was drawn by using the R package “ggplot2”. Subsequently, LASSO regression analysis was performed using the R package “glmnet”. The corresponding coefficients of M1 genes were assessed.

### Model validation

Response scores for all patients were generated using a response score model. Patients were divided into high response score and low response score groups based on the median response score. The Kaplan-Meier survival plot was generated by using R packages “survminer” and “survival.” The time-dependent receiver operating characteristic (ROC) plot was constructed by using R packages “timeROC” based on overall survival information.

### RT-qPCR analysis

Total RNA extraction of surgically excised tumor tissues, reverse transcription and RT-qPCR were performed as described previously [26], with *β-actin* as an internal control. The sequences of primers were shown in Table 2. The characteristics of TD-FOREKNOW advanced NSCLC patients were shown in Supplementary Table S2.

**Table:** **Table 2** Sequences of primers used for RT-PCR

Name	Sequence (5′→3′)
*SFTA3*-F	TGAGAGCCGGGTTTTCTGAC
*SFTA3*-R	CACAGGTGGGTATCCGCTTT
*NKX2-1*-F	CTCGCTCGCTCATTTGTTGG
*NKX2-1*-R	TCGGCGGCGGCTGAG
*CD8A*-F	CTCTCTGGACCCCGTTTCTG
*CD8A*-R	TGTGCCTGAATCAGCCTTTCT
*IL2RB*-F	ATGTCTCAGCCAGGGCTTC
*IL2RB*-R	GGCTCCAGACACAGGAGATG
*IDO1*-F	GCAAATGCAAGAACGGGACA
*IDO1*-R	GTTGCCTTTCCAGCCAGACA
*CXCL9*-F	TGATTGGAGTGCAAGGAACCC
*CXCL9*-R	AATTTTCTCGCAGGAAGGGCT
*β-actin*-F	CATCCGTAAAGACCTCTATGCCAAC
*β-actin*-R	ATGGAGCCACCGATCCACA

F, forward; R, reverse.

### Gene set enrichment analysis (GSEA)

The GSEA software (v.4.2.3) was downloaded from the website ( https://www.gsea-msigdb.org/gsea/index.jsp) [27]. Both the phenotype file and gene expression file were prepared in advance. “c2.cp.kegg.v2023.1.Hs.symbols.gmt” was set as the gene set database. The number of permutations selected was 1000, and other parameters were set as default. Finally, *P*<0.05 and FDR < 0.25 were considered to be statistically significant. The R package “GseaVis” was used for visualization.

### Drug sensitivity analysis

The R package “oncoPredict” was utilized to analyze drug sensitivity by training data from the Genomics of Cancer Drug Sensitivity (GDSC) database ( https://www.cancerrxgene.org/). The GDSC2 expression file and corresponding IC50 results were then downloaded from GDSC. The function “calcPhenotype()” was employed to compute the drug sensitivity of all analyzed samples.

### Statistical analysis

R software (version 4.1.2) was used to perform all the statistical analyses in this study, and *P*<0.05 was considered statistically significant.

## Results

### TME landscape in NSCLC patients

**Figure 1:**
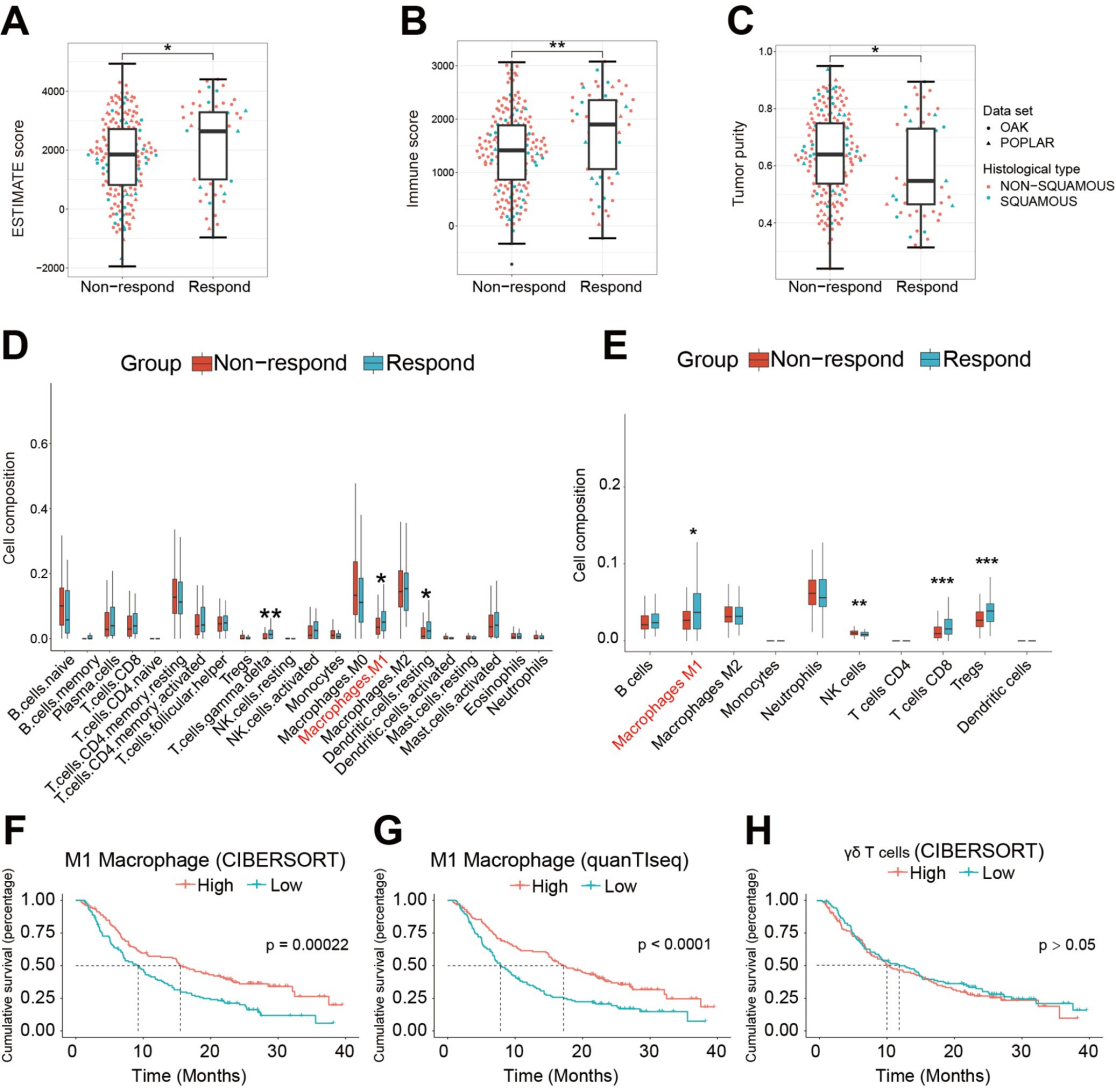
Figure 1 Tumor microenvironment landscape of advanced NSCLC (A‒C) Comparison of immune score (A), ESTIMATE score (B), and tumor purity (C) between nonresponders (progressive disease, n=193) and responders (complete response, partial response, n=61), shown for each histotype. Wilcoxon test, *P<0.05, **P<0.01. (D) Boxplot showing the infiltration of 22 immune cells evaluated by CIBERSORT between nonresponders and responders. Wilcoxon test, *P<0.05, ** P<0.01. (E) Boxplot showing the infiltration of 10 immune cells evaluated by quanTIseq between nonresponders and responders. Wilcoxon test, * P<0.05, **P<0.01, ***P<0.001. (F‒H) Kaplan-Meier survival curve of NSCLC patients in the training set grouped by M1 macrophage infiltration in CIBERSORT (F) and quanTIseq (G) and gamma delta T-cell infiltration (H).

Because the TME can significantly affect the overall immunotherapy response [28], we evaluated the composition of immune cells for each NSCLC patient. As shown in Figure 1A,B, the immune score and ESTIMATE score were significantly increased in the immunotherapy-responsive group in comparison with the immunotherapy-nonresponding group. Similarly, tumor purity was decreased in responders ( Figure 1C), suggesting that the responding group showed more immune cell infiltration. Thereafter, we measured immune cell infiltration by CIBERSORT, quanTIseq, and xCell. The results indicated that in comparison with the nonresponding group, the composition of gamma delta T cells and M1 macrophages was significantly enhanced in the responding group ( Figure 1D,E and Supplementary Figure S1). Compared with the nonresponding group, the composition of resting DC cells was enhanced, while the result of quanTIseq indicated that the composition of DCs in NSCLC was low. Similarly, CD4 ^+^ T cells, Tregs, and NK cells were enhanced in the responder group, while the CIBERSORT results indicated that these changes were not significant. To further demonstrate that high-level infiltration of certain types of immune cells enhance NSCLC prognosis, NSCLC patients were divided into a high infiltration group and a low infiltration group based on the infiltration of M1 macrophages and gamma delta T cells evaluated by CIBERSORT and quanTIseq. Kaplan-Meier survival curves showed that a higher level of M1 macrophages was significantly related to a promising prognosis and that infiltration of gamma delta T cells was not a prognostic predictor ( Figure 1F‒H). These results suggested that higher infiltration of M1 macrophages may be related to a favorable TME, which reinforces the immunotherapy effect.

### Screening out genes related to M1 macrophage infiltration

**Figure 2:**
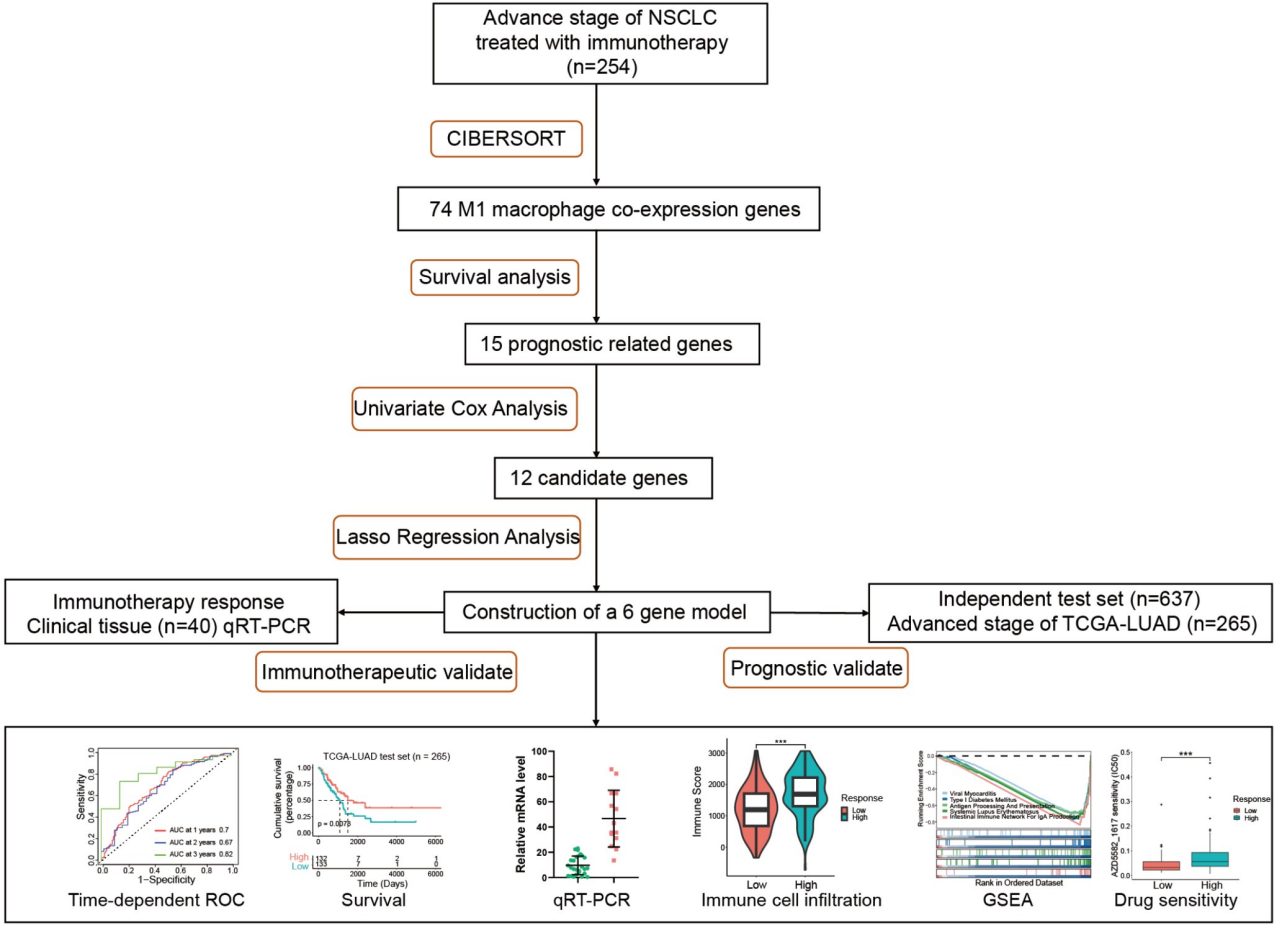
Figure 2 Research design RNA sequencing profiles for 1156 NSCLC patients are downloaded and CIBERSORT is performed to compute immune cell infiltration. Seventy four genes are screened out based on M1 macrophage infiltration and narrowed down to 6 genes by using Cox and Lasso regression analysis. Validation of prognostic and immunotherapeutic effect is conducted.

**Figure 3:**
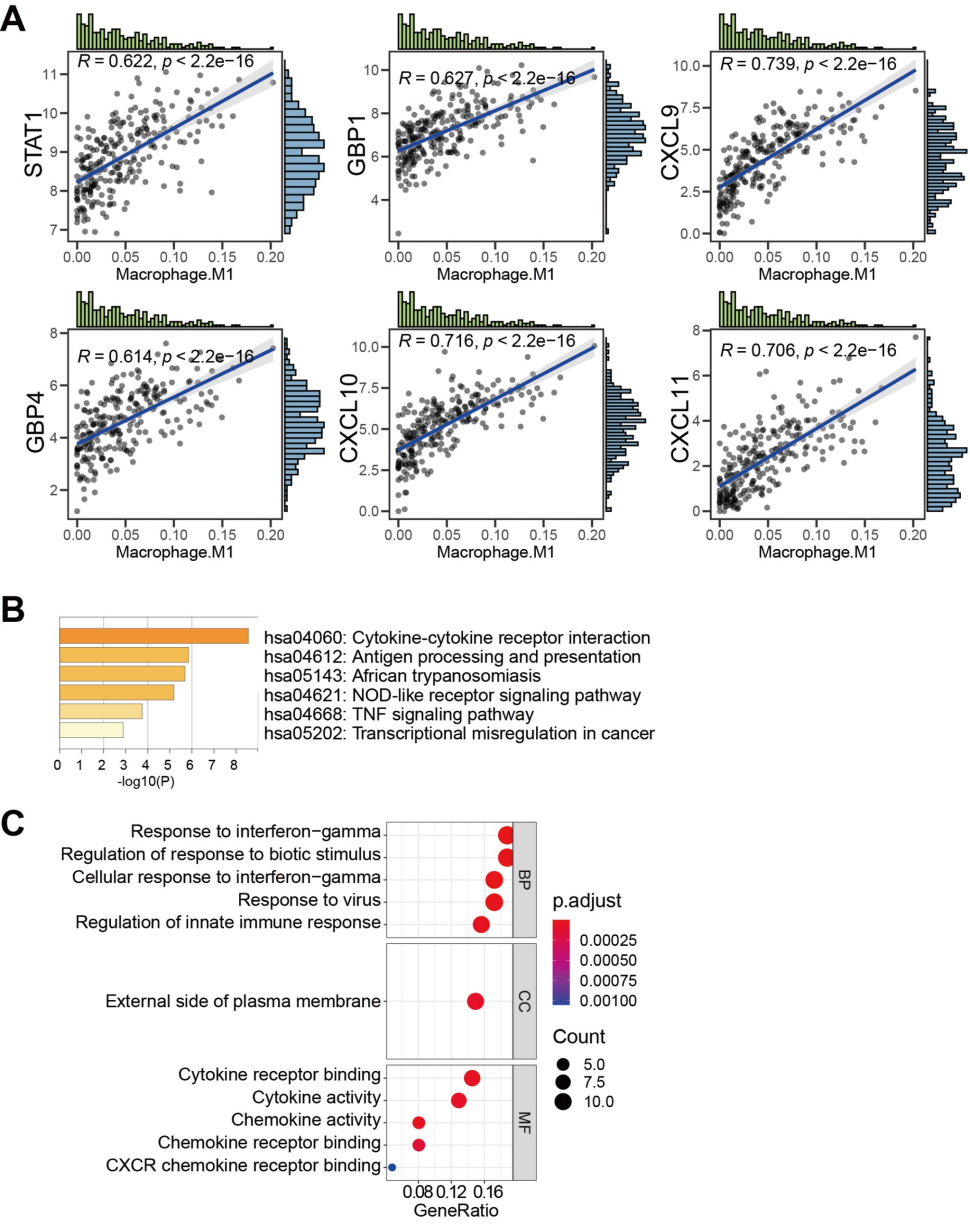
Figure 3 Screening of genes related to M1 macrophage infiltration (A) Correlation maps of the top 6 genes associated with M1 macrophage infiltration. (B) Bar graph of enriched KEGG terms across M1 macrophage-correlated genes, colored by P values. (C) Dot plot shows the results of GO analysis. BP, biological process, CC, cellular component, MF, molecular function.

Considering the potential role of M1 macrophages in the immunotherapy effect, this work aims to screen out M1 macrophage-related genes (M1 genes) and then construct an efficient model to predict the immunotherapy response for further precise NSCLC treatments ( Figure 2). M1 genes were screened from the expression profiles of the training dataset ( *n*=254 patients) by combining CIBERSORT. In total, 74 different genes showed a significant correlation with M1 macrophage infiltration ( Figure 3A), as *P*<0.01 and an absolute value of R>0.4 were set as thresholds.

To analyze the possible mechanism of these 74 M1 genes in M1 macrophage infiltration, we next investigated their functions by KEGG and GO enrichment analyses. KEGG analysis revealed that various M1 macrophage-related genes were mainly associated with cytokine-cytokine receptor interaction, antigen processing and presentation, African trypanosomiasis, nucleotide-binding domain (NOD)-like receptor signaling pathway, tumor necrosis factor (TNF) signaling pathway, and transcriptional misregulation in cancer ( Figure 3B). GO analysis showed that M1 macrophage-related genes were primarily related to response to interferon-gamma, regulation of response to biotic stimulus, external side of plasma membrane, and cytokine receptor binding ( Figure 3C). These results indicated that 74 M1 genes were involved in the regulation of the immune system to influence the immunotherapy response.

### Construction of immunotherapy response and prognostic model via M1 genes

**Figure 4:**
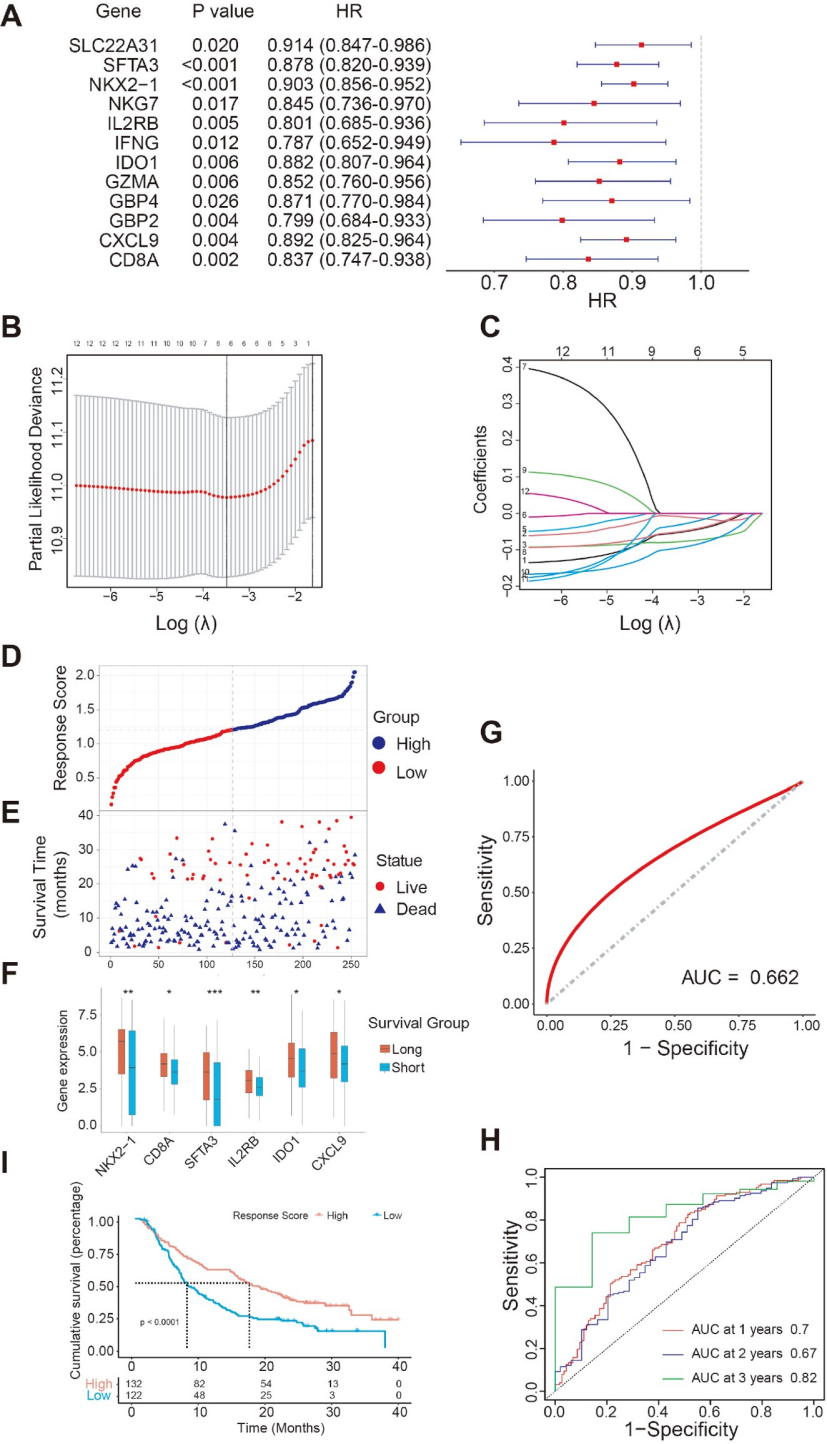
Figure 4 Construction of immunotherapy response and prognostic model via M1 genes (A) Forest plot shows the result of univariate Cox regression for overall survival. HR, hazard ratio. (B) Ten-fold cross-validation for tuning parameter selection in LASSO regression. Vertical lines are drawn from the best data according to the minimum criterion and 1 standard error criterion. (C) Variation curve of the regression coefficient with Log (λ) in LASSO regression. (D) Response score of patients in the training set arranged in ascending order. The gray dashed line shows the median cut-off value and divides the patients into a high response score group and a low response score group. (E) Distribution of survival time and status in the same order as in (D). (F) Expressions of six M1 genes between the long-survival group (n=117) and short-survival group (n=137) in the training set. Wilcoxon test, *P<0.05, **P<0.01, ***P<0.001. (G) Prediction value of the response score to immunotherapy response by ROC curve analysis (AUC=0.66, 95% CI=0.58‒0.74). AUC, area under the curve. (H) Time-dependent ROC curve analysis in the training set (AUC at 1 year=0.70, 95% CI=0.63‒0.76, AUC at 2 years=0.67, 95% CI=0.58‒0.76, AUC at 3 years=0.82, 95% CI=0.69‒0.94). (I) Kaplan-Meier survival curve of NSCLC patients in the training set.

To investigate whether M1 genes can be utilized to predict the immunotherapy response and prognosis of NSCLC patients, we performed a Kaplan-Meier survival analysis on 74 M1 genes. Among them, 15 genes were significantly related to either a significantly worse or better prognosis ( Supplementary Figure S2). Subsequently, univariate Cox proportional regression was conducted on 15 previously screened genes, which led to narrowing down to 12 genes: *NKX2-1*, *CD8A*, *SFTA3*, *IL2RB*, *IFNG*, *GZMA*, *GBP4* , *IDO1*, *SLC22A31*, *CXCL9*, *GBP2* and *NKG7* ( Figure 4A). Thereafter, to construct an optimal model, we conducted LASSO regression analysis on 12 candidate genes. The lambda value was set at the minimum, and finally, 6 genes were shortlisted: *CD8A*, *CXCL9*, *NKX2-1*, *SFTA3*, *IDO1*, and *IL2RB* ( Figure 4B,C). Next, a calculation formula of the model score based on the six M1 genes was calculated based on their regression coefficients through multivariate Cox analysis: response score=( *NKX2-1* exp×0.053)+( *CD8A* exp×0.008)+( *SFTA3* exp×0.078)+( *IL2RB* exp×0.032)+( *IDO1* exp×0.050)+( *CXCL9* exp×0.090). Thus, response scores were calculated based on M1 genes in the training dataset ( *n*=254 patients). Then, patients were divided into high-score and low-score groups based on the median cut-off response score ( Figure 4D). Figure 4E illustrated that patients with a high response score had a longer survival time and favorable survival status. The expression of the six M1 genes showed different expression patterns between the long survival group (survival time longer than 1 year) and the short survival group (survival time shorter than 1 year) ( Figure 4F). To test the efficiency for predicting immunotherapy response, a ROC curve was constructed, and the area under the curve (AUC) was 0.66 (95% CI: 0.58‒0.74) ( Figure 4G), indicating a fine predictive value for immunotherapy response in NSCLC. Moreover, the accuracy was verified by time-dependent ROC curves, and the AUC values were 0.7 at 1 year, 0.67 at 2 years, and 0.82 at 3 years, indicating high prediction specificity and sensitivity ( Figure 4H). Additionally, survival analysis showed that the high response score group had a favorable prognosis ( *P* <0.0001; Figure 4I). These results indicated that the response score computed by the model based on the M1 genes may be utilized to predict NSCLC immunotherapy response and prognosis with credible accuracy.

### Validation of model prognostic efficiency in the independent test set

**Figure 5:**
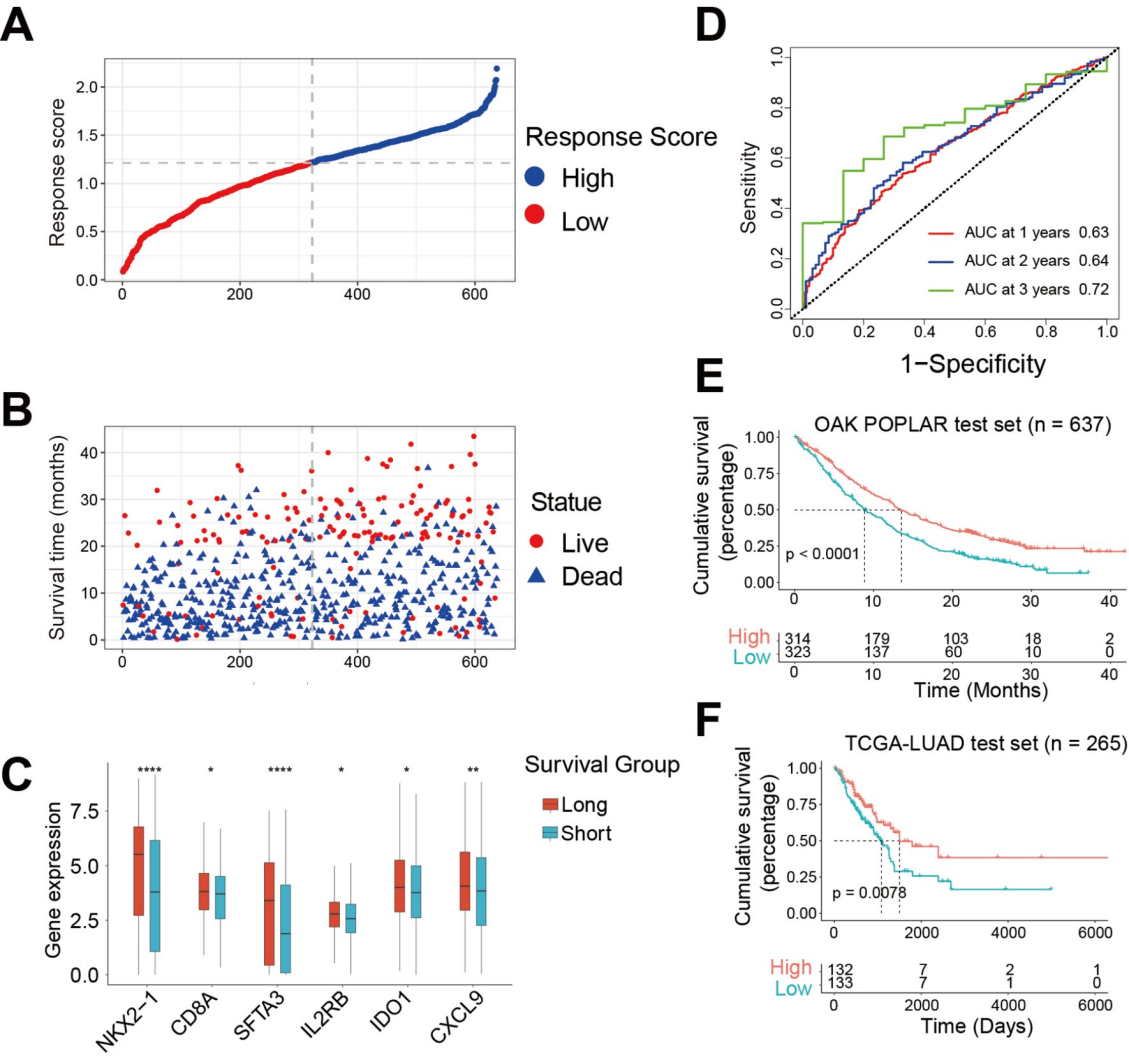
Figure 5 Validation of model prognostic efficiency in the independent test set (A) Response score of patients in the internal test set (n=637) arranged in ascending order. The gray dashed line shows the median cut-off value and divides the patients into a high response score group and a low response score group. (B) Distribution of survival time and status in the same order as in (A). (C) Expressions of 6 M1 genes between the long-survival group (n=281) and short-survival group (n=356) in the test set. Wilcoxon test, *P<0.05, **P<0.01, ***P<0.001, ****P<0.0001. (D) Time-dependent ROC curve analysis in the internal test set (n=637) (AUC at 1 year=0.63, 95% CI=0.58‒0.67, AUC at 2 years=0.64, 95% CI=0.58‒0.70, AUC at 3 years=0.72, 95% CI=0.62‒0.82). (E,F) Kaplan-Meier survival curve of NSCLC patients in the internal test set (n=637) (E) and external independent TCGA test (n=265) (F) cohort.

To assess our model’s predictive accuracy, we computed the response score in the test set ( *n*=637) and divided the test set into a low response score group and a high response score group based on the median cut-off value ( Figure 5A). We found that the survival of NSCLC patients was significantly improved as the model score increased, and the six M1 genes showed different expression patterns between the long survival time group and the short survival group ( Figure 5B,C). Moreover, the accuracy was verified by time-dependent ROC curves, and the AUC values were 0.65, 0.66, and 0.76 at 1, 2 and 3 years, respectively ( Figure 5D). Consistently, survival analysis indicated a better prognosis in the high response score group ( Figure 5E). Next, we validated the model in an external independent TCGA-LUAD set. Kaplan-Meier survival analysis showed that the high response score group had a better prognosis than the low response score group ( Figure 5F). Overall, the above results in both the internal and external independent test sets indicated that the model indeed had excellent performance in predicting NSCLC prognosis.

### Efficiency of M1 genes for immunotherapy response prediction

**Figure 6:**
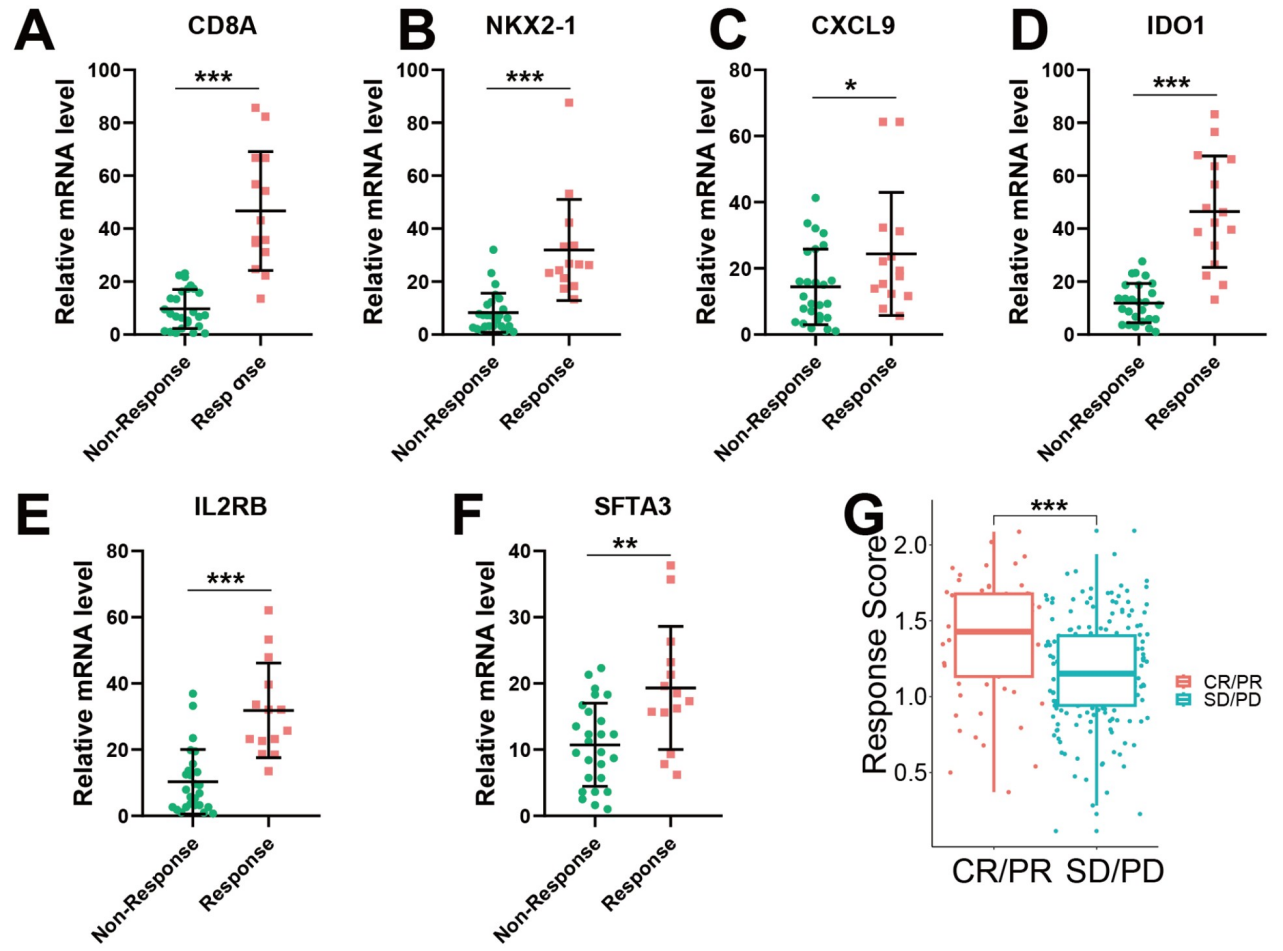
Figure 6 Efficiency of M1 genes for immunotherapy response prediction (A‒F) Validation of the expressions of M1 genes between the immunotherapy nonresponse and response groups by RT-qPCR from the TD-FOREKNOW NSCLC clinical trial (n=40). (A) CD8A. (B) NKX2-1. (C) CXCL9. (D) IDO1. (E) IL2RB. (F) SFTA3. Wilcoxon test, *P<0.05, **P<0.01, *** P<0.001. (G) Correlation between response scores and clinical response to immunotherapy. PD, disease progression, SD, disease stable, PR, partial response, CR, complete response. Wilcoxon test, *P<0.05, **P<0.01, ***P<0.001.

To verify the efficacy of six M1 genes in predicting real-world neoadjuvant immunotherapy plus chemotherapy response, the TD-FOREKNOW randomized clinical trial (ClinicalTrials.gov Identifier: NCT04338620) enrolled 43 patients with advanced stage NSCLC and administered neoadjuvant camrelizumab plus chemotherapy (nab-paclitaxel and platinum) for 3 cycles as we previously reported [5]. Four to six weeks after administration, 40 of the 43 patients underwent surgery, and the other 3 patients did not receive surgery. Surgical specimens of the primary tumor and all sampled local lymph nodes without viable tumor cells were considered responsive to canrezumab plus chemotherapy. Conversely, they were considered nonresponders. The expressions of *CD8A*, *CXCL9*, *NKX2-1*, *SFTA3*, *IDO1*, and *IL2RB* in the response group was markedly higher, suggesting that six M1 genes were indeed involved in NSCLC sensitivity to camrelizumab plus chemotherapy ( Figure 6A‒F). Moreover, the response score in the response group was also significantly higher than that in the nonresponse group, indicating that this response score model can be utilized to predict NSCLC sensitivity to camrelizumab plus chemotherapy ( Figure 6G).

### Response score reveals the TME and TIM in NSCLC

**Figure 7:**
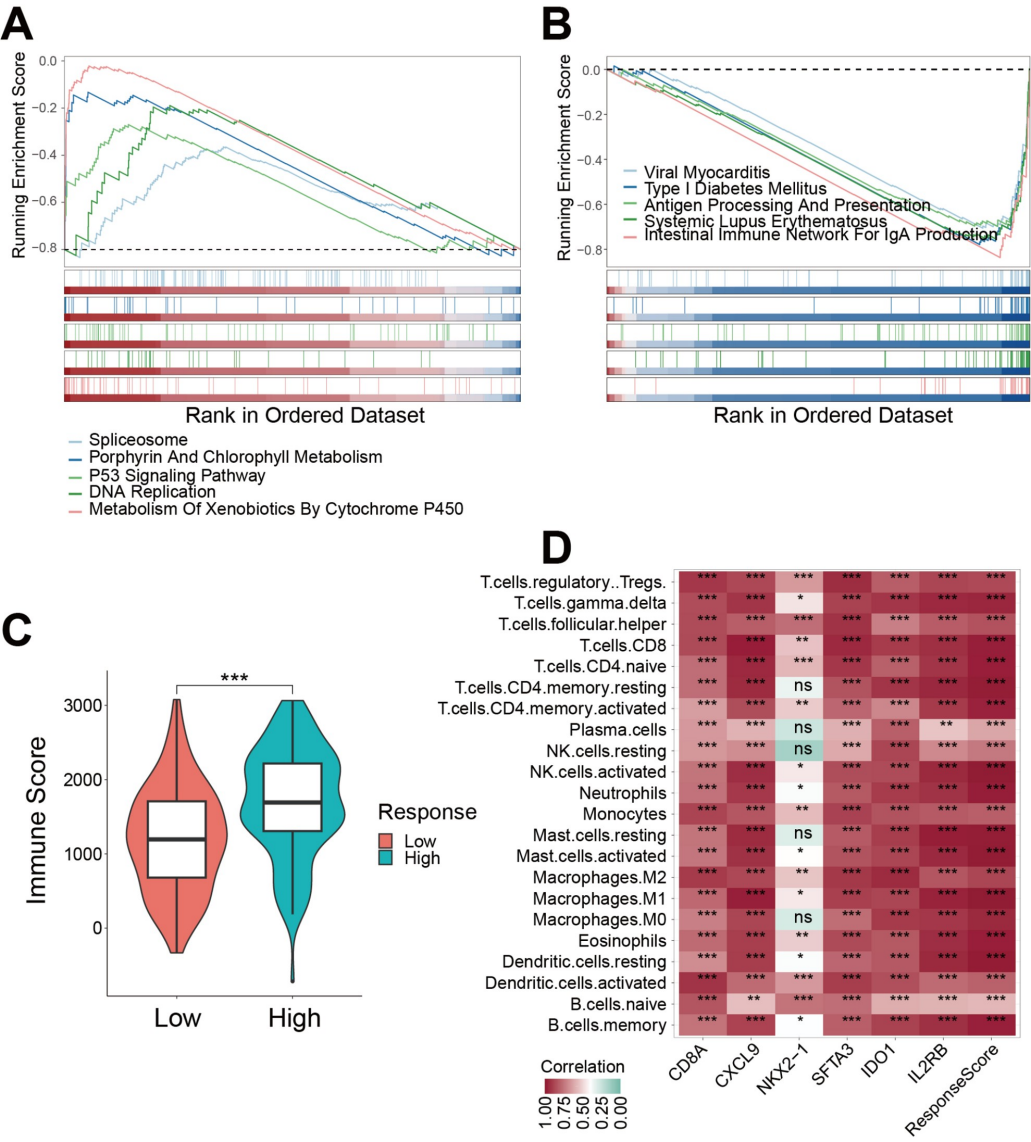
Figure 7 Response score reveals tumor microenvironment and tumor immune microenvironment in NSCLC (A,B) Results of GSEA enrichment pathway analysis in the low-response score (A) and high-response score (B) groups. (C) Violin plot showing the different immune scores between the low-response and high-response score groups. Wilcoxon test, *P<0.05, **P<0.01, ***P<0.001. (D) Heatmap shows the correlation between M1 genes and response score with 22 immune cell infiltration. *P<0.05, **P<0.01, *** P<0.001.

Given that the response score model based on six M1 genes showed excellent efficacy in predicting immunotherapy sensitivity and prognosis, GSEA was further carried out to explore the potential mechanisms influencing immunotherapy response and prognosis ( Supplementary Table S3). The various enrichment pathways of the low response score group include spliceosome, porphyrin, and chlorophyll metabolism, P53 signaling pathway, DNA replication, and metabolism of xenobiotics by cytochrome P450 ( Figure 7A). Enrichment pathways identified in the high response score group were those related to viral myocarditis, type I diabetes mellitus, antigen processing and presentation, systemic lupus erythematosus, and intestinal immune network for IgA production ( Figure 7B). These observations suggested that the high response score group may have a better prognosis and improved response to immunotherapy through these possible mechanisms.

It was noted that the immune score was significantly increased in the high response score group compared with that in the low response score group, suggesting that this prediction model may identify the immune cell infiltration level ( Figure 7C). Indeed, the expression of six M1 genes and response score were significantly correlated with increased levels of memory B cells, macrophages, resting mast cells, monocytes, activated CD4 memory T cells, and resting NK cell infiltration ( Figure 7D). These results suggested that high-level infiltration of CD4 T cells, B cells, mast cells, NK cells, and macrophages could form a favorable TME that could be correlated with a better prognosis through the mechanisms mentioned above.

### Results of drug sensitivity analysis

**Figure 8:**
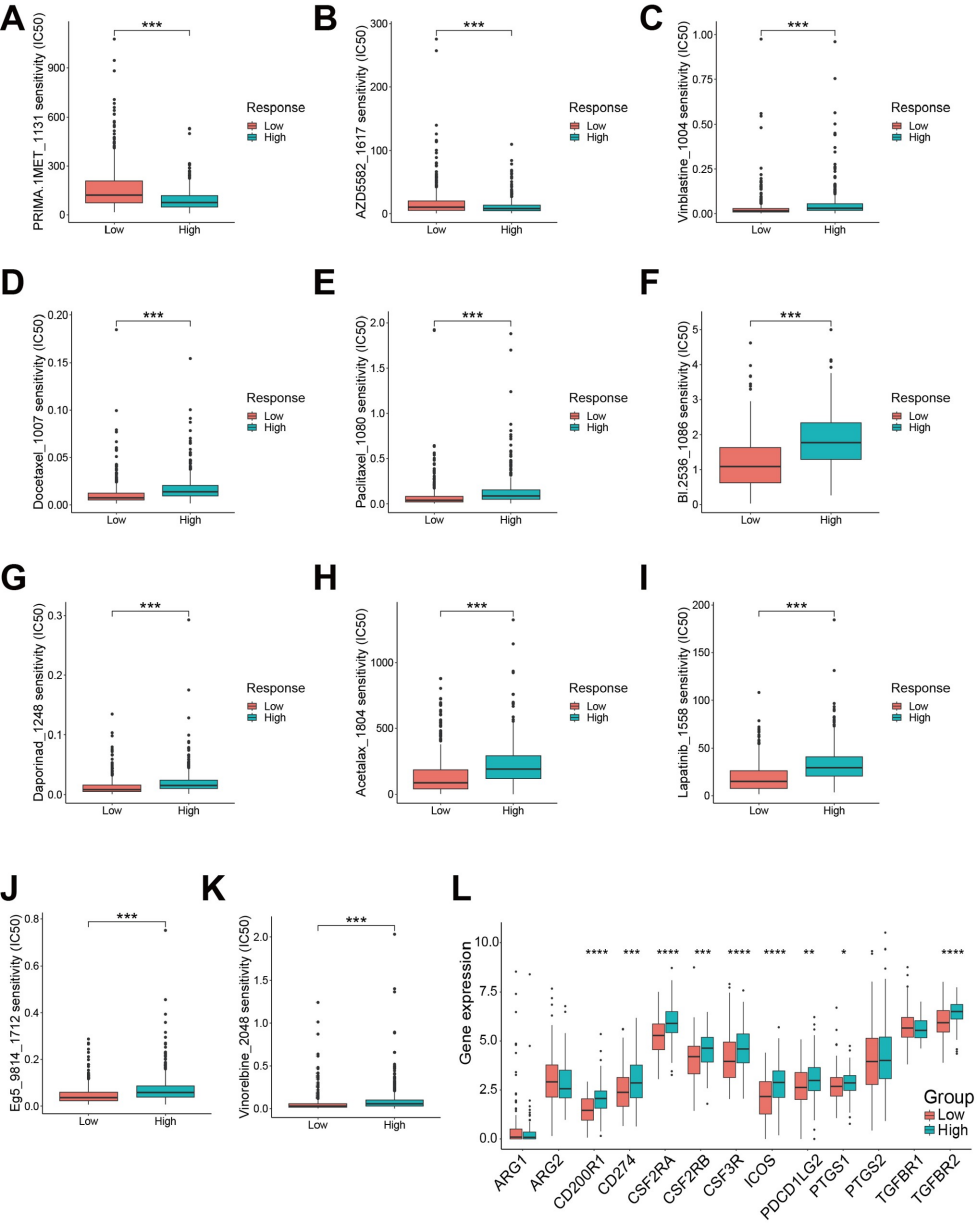
Figure 8 Results of drug sensitivity analysis (A‒K) Different IC50 values of target or chemotherapy drugs between the low response score and high response score groups. (A) PRIMA-1MET. (B) AZD5582. (C) Vinblastine. (D) Docetaxel. (E) Paclitaxel. (F) BI-2536. (G) Daporinad. (H) Acetalax. (I) Lapatinib. (J) Eg5_9814. (K) Vinorelbine. (L) Immune checkpoint gene expression between the low response score and high response score groups. Wilcoxon test, * P<0.05, **P<0.01, ***P<0.001, ****P<0.0001.

As previously described, the response score can be utilized to predict the drug sensitivity of immunotherapy ( Figure 6G). Next, the sensitivity of 198 drugs in the 891 NSCLC patients was studied to evaluate the correlation between the response score and sensitivity of drugs ( Supplementary Table S4). Specifically, the high response score group exhibited a lower IC _50_ value for the two targeted drugs, namely, PRIMA-1MET and AZD5582 ( Figure 8A,B). Moreover, nine other chemical or targeted drugs were identified to exhibit a lower IC _50_ values in the low response score group, including vinblastine, docetaxel, paclitaxel, BI-2536, daporinad, acetalax, vinorelbine, lapatinib (targeting HER2) and Eg5_9814 (targeting KSP11), suggesting that the low response score group may benefit from these selected drugs ( Figure 8C‒K). Furthermore, the immune checkpoints *CD200R1*, *CD274*, *CSF2RA*, *CSF2RB*, *CSF3R* , *ICOS*, *PDCD1LG2*, *PTGS2*, and *TGFBR2* were expressed at higher levels in the high response score group, yet *ARG1* and *ARG2* were expressed at higher levels in the low response score group ( Figure 8L). These results provide novel insights into NSCLC ICI treatment selection. Based on these findings, this response model can aid in further selection of not only immunotherapy but also chemotherapy and enhance the precision and efficacy of drug therapy.

## Discussion

Due to the lack of early symptoms and reliable biomarkers in NSCLC, therapy options and prognosis are substantially compromised [29]. In this study, we constructed a novel immunotherapy response prediction model based on M1 macrophage-related genes. M1 macrophages can reduce lung cancer cell migration in the TME and boost the effectiveness of anti-PD1 immunotherapy, as reported previously [ 30, 31]. Similarly, we found that better immunotherapy outcomes were directly related to higher levels of M1 macrophage infiltration. Additionally, the response score computed by our model revealed the TME landscape of NSCLC and can be used for immunotherapy response prediction, individualized sensitive drug selection and prognosis evaluation. Specifically, high response score group patients displayed a favorable prognosis following high-level infiltration of NK cells, CD4 ^+^ T cells, B cells, mast cells, and macrophages through the orchestration of the TME. Moreover, the high response score group benefited more from immunotherapy than the low response score group. In contrast, the low response score group exhibited a poor prognosis and was unlikely to respond to immunotherapy, but we observed that the targeted drugs lapatinib and Eg5_9814 may benefit the low response score group more. Collectively, our model can serve as a novel tool for clinicians to evaluate immunotherapy response and prognosis and further make personalized treatment decisions for NSCLC patients. Furthermore, in comparison to a large number of gene expression profiles, our approach only needs to identify the expression of six hub genes, which can significantly minimize the operational cost and methodological bias and increase its applicability in the clinic [32].

In this study, 6 hub genes, *NKX2-1*, *CD8A*, *SFTA3*, *IL2RB*, *IDO1*, and *CXCL9*, which are all M1 macrophage coexpressed genes, were utilized to build the immunotherapy response model. *NKX2-1* can regulate lung cancer growth by targeting the MAPK pathway [33]. Importantly, this mechanism, together with lapatinib’s increased sensitivity in the low response score group, is similar to a previous study that identified human epidermal growth factor receptor 2 (HER2) and MAPK to be clinically significant molecular targets [34]. Surfactant-associated protein 3 (SFTA3) functions as a key immune system regulator in the respiratory system, thus providing critical protection against inflammation at mucosal sites [35]. *CD8A* and *IL2RB* expression levels are markedly higher in advanced lung adenocarcinoma, the most prevalent histological type of NSCL, indicating its predictive function in NSCLC [ 36, 37]. Tryptophan (Trp), an essential amino acid, is converted into downstream kynurenines by the rate-limiting metabolic enzyme indoleamine 2,3-dioxygenase 1 (IDO1) which has been identified as a potential immunotherapy target [38]. Interestingly, using transcriptomic analysis, Garrido *et al* [39] reported that M1-like tumor-associated macrophages can recruit and sustain tissue-resident memory T cells through *CXCL9* expression to augment adaptive antitumor responses. Additionally, previous studies have also demonstrated that TAM *CXCL9* expression could increase the anti-PD (L)-1 response rate through positioning stem-like CD8 T cells [40]. In conclusion, the M1 genes identified in this study could be used as immunotherapy targets in the future. Next, we will conduct further experiments on these M1 genes to uncover the various underlying pathogenic processes in NSCLC.

To assess the model’s efficacy and accuracy in prognosis prediction, 1-, 2-, and 3-year ROC curves were generated in the training set, and the corresponding AUC values were found to be near 0.7. Kaplan-Meier survival analysis in an independent TCGA-LUAD set was also performed, showing an outstanding predictive effect in predicting the prognosis of advanced-stage NSCLC. Li *et al*. [41] previously reported a 28-gene signature in TCGA-NSCLC patients with a 3-year AUC of 0.69 in the training dataset, which was lower than the AUC observed in our analysis despite involving more genes. In this study, we found that the response score was significantly associated with immunotherapy response. The expressions of these six M1 genes showed significant differences in real-world clinical advanced-stage NSCLC patients. These results clearly demonstrated the crucial therapeutic importance of our model and prompted us to look into the potential underlying processes.

Considering the critical role of TME in tumor progression, patient prognosis, and immunotherapy response [42], we first analyzed the TME landscape of advanced-stage NSCLC patients after administration of immunotherapy and then determined the discrepancy level of immune cell infiltration. Specifically, M1 macrophages showed higher infiltration in responders than in nonresponders, which was consistent with the discovery of Gunassekaran *et al* . [43] showing that a high degree of M1-like macrophage infiltration can significantly boost antitumor immunity. In addition, TAMs in the high response score group showed higher infiltration levels computed using our model. Previous studies have indicated that Fc receptors of TAMs can bind to the Fc portion of antibodies, which leads to antibody internalization or blockade of their interaction with immune cells [ 44, 45]. Similarly, enrichment analysis revealed that M1 genes can influence the immunotherapy response by regulating the immune system. Furthermore, the possible mechanism underlying the response score computed by the model predicting the immunotherapy response of advanced-stage NSCLC patients was studied by using GSEA. The results of the high- vs. low-response score group were in agreement with previous reports that *P53* inactivation was strongly related to tumorigenesis and treatment resistance [46]. Overall, our model demonstrated a significant predictive value of immunotherapy in advanced NSCLC. The potential biological mechanisms could be attributed to the interference with the function of therapeutic antibodies by TAM and *P53* activation in TAM.

Immunotherapy has drastically altered lung cancer treatment during the last few decades [47]. However, immunotherapeutic drugs have been found to be ineffective in most NSCLC patients [48]. Thus, discovering accurate biomarkers to effectively improve the response rate of immunotherapy in NSCLC is critical. Our study focused on advanced-stage NSCLC patients treated with ICIs whose immunotherapy response was validated in clinical settings, thus representing the real world of NSCLC treatment. In this work, we first showed that the response score can reliably predict the outcome of immunotherapy. The expressions of M1 genes were validated in clinical tissue by RT-qPCR. In a previous study, based on T-cell dysfunction and exclusion, Jiang *et al*. [49] developed the TIDE algorithm with the intention of predicting immunotherapeutic outcomes, which has been widely used in various cancers. Moreover, a recent study reported that macrophages can exhibit complex interactions with T cells, including macrophage-mediated T-cell elimination, inhibition and exhaustion by recruiting myeloid-derived suppressor cells and regulatory T cells, and upregulation of *PD-L1*, *arginase*, and other exhaustion-related genes [ 14, 50‒ 52]. However, unlike TIDE, the M1 macrophage coexpression gene-based model we developed showed both immunotherapeutic prediction value and excellent prognostic prediction value. Furthermore, our findings revealed that targeted drugs such as lapatinib (targeting HER2) and Eg5_9814 (targeting KSP11) were more sensitive in the low-response group than in the high-response score group. Interestingly, the expressions of *arginase-1* ( *ARG1*) and *ARG2* were significantly higher in the low-response group, suggesting that they could be used as therapeutic targets. These findings could play a significant role in therapy selection in immunotherapeutic nonresponding NSCLC patients, and they establish the need for novel drugs to improve prognosis. In fact, HER2-targeted therapies have demonstrated substantial anticancer activity [53]. The function and mechanism of novel immunotherapy targets and agents for NSCLC require more research.

Nevertheless, there are still a few limitations in our study. We constructed the model based on only 254 NSCLC patients, and a merely three-year follow-up period was allotted because the patients were diagnosed at an advanced stage. Further long-term studies are urgently needed, and identification of NSCLC at an early stage needs a better prognostic model which we intend to develop in the future. More independent experimental research is also needed to clarify the molecular pathways by which the M1 genes influence NSCLC and to confirm the accuracy of our model.

In conclusion, we constructed a novel M1 macrophage coexpression gene model that serves as both an effective prognostic and predictive biomarker that can provide information related to the effectiveness of immunotherapy in NSCLC. Importantly, the response score computed by our model was significantly correlated with the TME and drug susceptibility, suggesting the involvement of mechanisms associated with tumor progression. Future clinical research is also needed to validate our approach.

## Supporting information

23406Supplementary_Figures

Supplementary_table_S1_Charactristics_of_Advanced_Lung_Adenocarcinoma_Patients_in_TCGA-LUAD_Project

Supplementary_table_S2_Characteristics_of_TD-FOREKNOW_Advanced_Non_Small_Cell_Lung_Cancer_Patients

Supplementary_table_S3_Result_of_GESA_Analyse_Between_High_Response_Score_Group_and_Low_Response_Score_Group

Supplementary_table_S4_Result_of_Drug_Sensitivity_Prediction_in_Training_Set_Patients

## Supplementary Data

Supplementary data is available at *Acta Biochimica et Biophysica Sinica* online.
