# Supplementary material for: M1 macrophage-related gene model for NSCLC immunotherapy response prediction: Gene model for NSCLC immunotherapy response prediction
Source: Acta Biochim Biophys Sin (Shanghai). 2024 Feb 21;56(3):379–92. doi: 10.3724/abbs.2023262 (PMC10984861; doi:10.3724/abbs.2023262)
Supplement: 23406Supplementary_Figures [file 23406Supplementary_Figures.pdf]

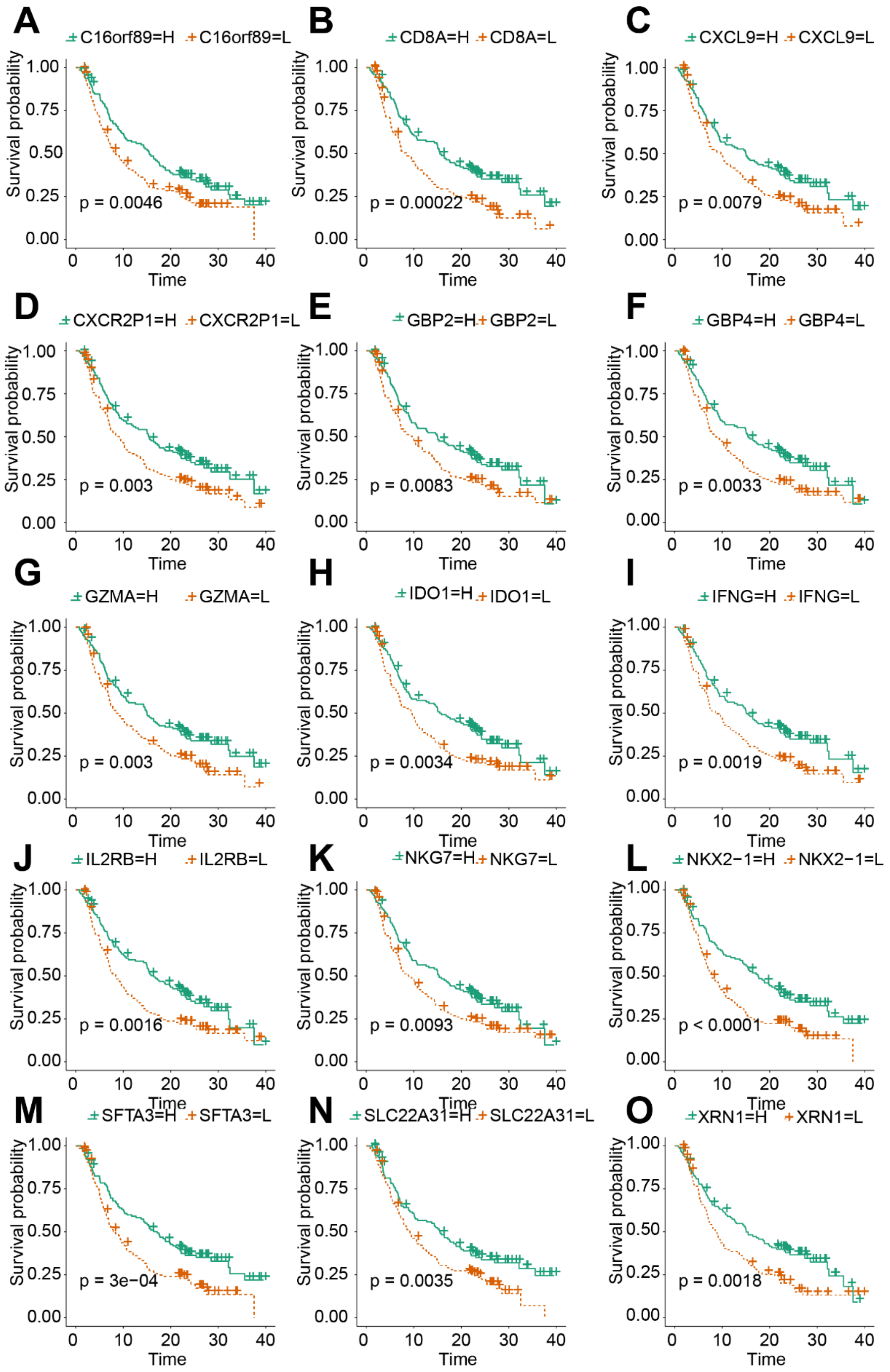

Supplementary Figure S1. Boxplot showing the immune cell infiltration evaluated by xCell between nonresponders and responders Wilcoxon test, **P*<0.05, ***P*<0.01, ****P*<0.001, *****P*<0.0001.

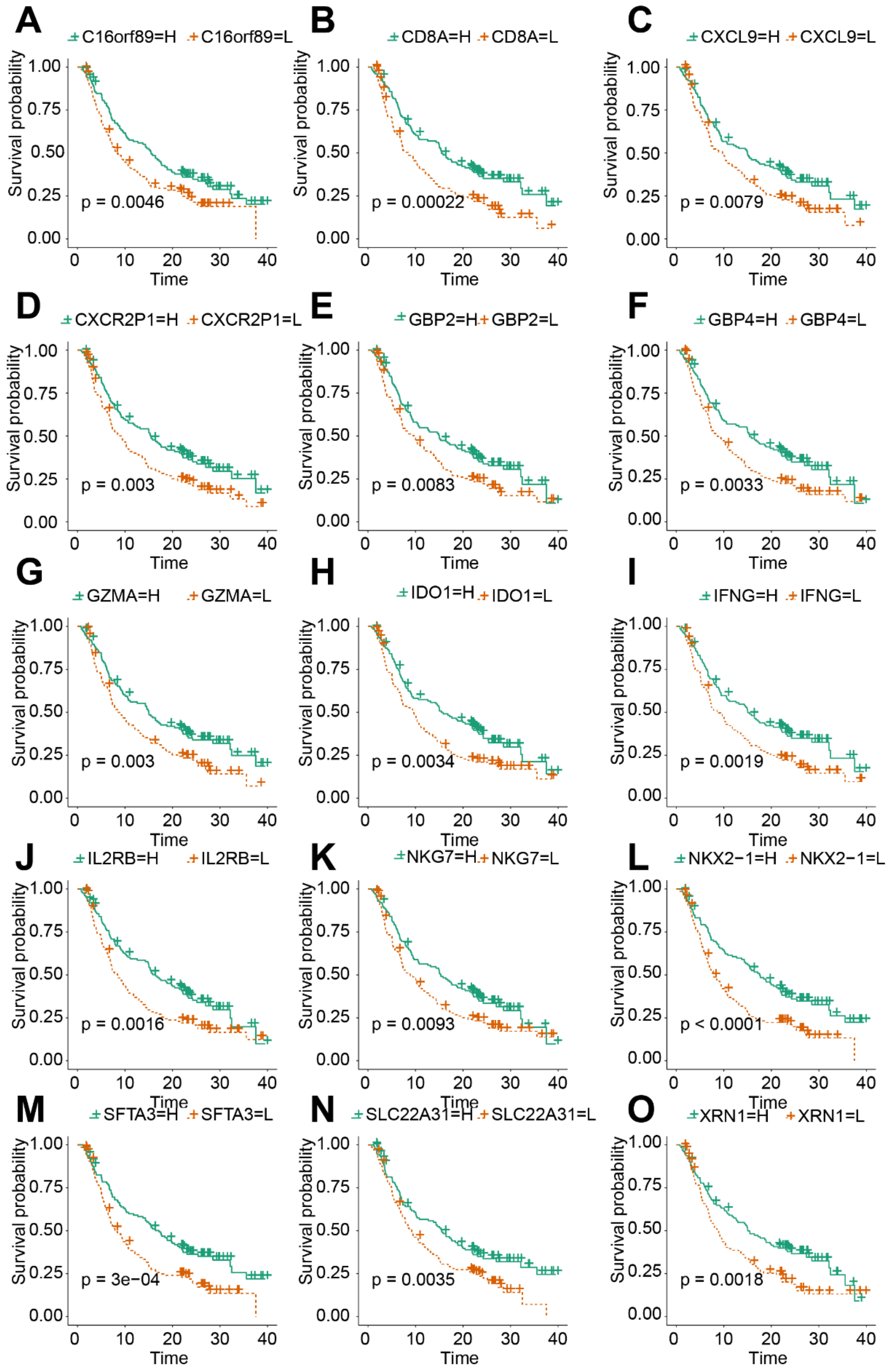

Supplementary Figure S2. Identification of prognostic M1 genes in NSCLC from the training set Kaplan-Meier curves for the 15 prognostic M1 genes, including (A) *C16orf89*, (B) *CD8A*, (C*)* *CXCL9*, (D) *CXCR2P1*, (E) *GBP2*, (F)

GBP4, (G) *GZMA*, (H) *IDO1*, (I) *IFNG*, (J) *IL2RB*, (K) *NKG7*, (L) *NKX2-1*, (M) *SFTA3*, (N) *SLC22A31*, and (O) *XRN1*. H: high-expression group; L, low-expression group.
